# Retraction: Chemopreventive Evaluation of a Schiff Base Derived Copper (II) Complex against Azoxymethane-Induced Colorectal Cancer in Rats

**DOI:** 10.1371/journal.pone.0328986

**Published:** 2025-07-28

**Authors:** The PLOS One Editors

After this article [1] was published, concerns were raised regarding results presented in Figs 1 and S1.

Specifically:

- The following panels appear to partially overlap:
  - Fig 1D in [1] and Fig 5B in [2]
  - Fig S1C in [1] and Fig 4E in [3, corrected in 4 and retracted in 5]
- Fig 1B in [1] appears similar to Fig 5B in [3, corrected in 4 and retracted in 5]

In response to the above concerns, a co-author and the corresponding author stated that they were unable to retrieve the original data underlying the results in this study, and the co-author requested retraction of the article.

In light of the above concerns which question the reliability of the reported results and conclusions, the *PLOS One* Editors retract this article.

SZM and NAM agreed with the retraction. NAM apologized for the issues with the published article. MAA did not respond to the final editorial decision. MH, PH, NSG, SG, ANH, AAA, MZ, ER, HK, SF, BK, and HMA either did not respond directly or could not be reached.
